# An Updated Perspective on *Sinorhizobium meliloti* Chemotaxis to Alfalfa Flavonoids

**DOI:** 10.3389/fmicb.2020.581482

**Published:** 2020-10-23

**Authors:** K. Karl Compton, Sherry B. Hildreth, Richard F. Helm, Birgit E. Scharf

**Affiliations:** ^1^Department of Biological Sciences, Life Sciences I, Virginia Tech, Blacksburg, VA, United States; ^2^Department of Biochemistry, Fralin Life Science Institute, Virginia Tech, Blacksburg, VA, United States

**Keywords:** motility, symbiosis, plant-host interaction, rhizosphere, solid-phase extract

## Abstract

The symbiotic interaction between leguminous plants and their cognate rhizobia allows for the fixation of gaseous dinitrogen into bioavailable ammonia. The perception of host-derived flavonoids is a key initial step for the signaling events that must occur preceding the formation of the nitrogen-fixing organ. Past work investigating chemotaxis – the directed movement of bacteria through chemical gradients – of *Bradyrhizobium japonicum*, *Rhizobium leguminosarum*, and *Rhizobium meliloti* discovered chemotaxis to various organic compounds, but focused on chemotaxis to flavonoids because of their relevance to the symbiosis biochemistry. The current work sought to replicate and further examine *Sinorhizobium* (*Ensifer*) *meliloti* chemotaxis to the flavonoids previously thought to act as the principal attractant molecules prior to the initial signaling stage. Exudate from germinating alfalfa seedlings was analyzed for composition and quantities of different flavonoid compounds using mass spectrometry. The abundance of four prevalent flavonoids in germinating alfalfa seed exudates (SEs) was at a ratio of 200:5:5:1 for hyperoside, luteolin, luteolin-7-glucoside, and chrysoeriol. Using quantitative chemotaxis capillary assays, we did not detect chemotaxis of motile *S. meliloti* cells to these, and two other flavonoids identified in seed exudates. In support of these findings, the flavonoid fraction of seed exudates was found to be an insignificant attractant relative to the more hydrophilic fraction. Additionally, we observed that cosolvents commonly used to dissolve flavonoids confound the results. We propose that the role flavonoids play in *S. meliloti* chemotaxis is insignificant relative to other components released by alfalfa seeds.

## Introduction

Plants of the Fabaceae family share a unique association with specific soil-dwelling bacteria that allows the plants access to the otherwise unavailable nitrogen in the atmosphere. This association with their cognate bacterial symbionts, referred to as rhizobia, has precipitated a great diversification of species in this family, yielding some of the most important crops in agriculture (Brewin, 1991; van Rhijn and Vanderleyden, 1995; Sprent, 2007).

Over the last several decades, the molecular basis for the development of this interaction has been rigorously expounded. The first step initiating this symbiosis is the release of flavonoids by the plant. Flavonoids are phenylpropanoid derivatives and act as signaling molecules, antimicrobials, and growth promoters (Waage and Hedin, 1985; Peters et al., 1986; Peters and Long, 1988; Hartwig et al., 1990a, 1991; Wachter et al., 1999; Sato et al., 2000). Rhizobia receive these flavonoid signals using the sensor protein NodD, which, subsequent to proper flavonoid binding, will induce the expression of “*nod*-box genes” (Peters et al., 1986; Hartwig et al., 1990a,b; Peck et al., 2006). Each rhizobial species may have one or several NodD copies, each with a unique specificity for certain flavonoids (Fisher and Long, 1992; Phillips et al., 1992). While a single flavonoid is sufficient to induce *nod* gene expression, rhizobia are exposed to a cocktail of host-derived flavonoids (Maxwell et al., 1989; Hartwig et al., 1990a; Maxwell and Phillips, 1990). Within a species, the makeup of this cocktail varies dramatically depending on the growth stage, location, and status of the plant (Maxwell et al., 1989; Maxwell and Phillips, 1990).

Flavonoids are not the only host-derived compounds that can affect *nod* gene expression. For example, trigonelline and stachydrine from *Medicago* spp. can induce *nod* gene expression, while the isoflavonoid-derivative medicarpin and the phytoestrogen coumestrol are both antagonists of *nod* gene expression (Phillips et al., 1992; Zuanazzi et al., 1998). The total involvement of these compounds in the symbiotic interaction is incompletely understood. Among the suite of genes induced by NodD is those involved in the synthesis of Nod factors, which are lipo-chitooligosaccharides that reciprocate the symbiotic signal to the plant. Reception of Nod factors occurs at the root hair. Among the most salient changes are the curling of the root hair around the population of rhizobia and the formation of the infection thread (IT), an invagination of the plant cell that will traverse into the cortical cells of the root. The rhizobia occupy the IT as it makes its way to what will become nodule primordia. The IT will ramify and act to hold a population of rhizobial cells that will terminally differentiate into nitrogen-fixing bacteroids once seeded into nodule cells (Brewin, 1991; van Rhijn and Vanderleyden, 1995; Wang et al., 2012; Haag et al., 2013; Udvardi and Poole, 2013).

Prior to all the above, the rhizobia must localize to the tips of developing root hairs. Because the nodule is a highly sought-after niche, there is a strong competition among rhizobia that can successfully colonize the host (Pinochet et al., 1993; Hirsch and Spokes, 1994; Da and Deng, 2003). While the flagella-driven motility and chemotaxis system has been shown to be unnecessary for nodule formation and nitrogen fixation, motility and chemotaxis provide a competitive advantage in all investigated rhizobia-host symbioses. In the rhizosphere, locomotion and navigation create the ability to out-compete neighbors for nodule occupancy (Ames et al., 1980; Napoli and Albersheim, 1980; Ames and Bergman, 1981; Miller et al., 2007; Lacal et al., 2010; Scharf et al., 2016). Motility and chemotaxis are critical for bacteria that occupy multiple niches or need to seek out spatial niches (Lacal et al., 2010). Thus, chemotaxis systems are ubiquitous in numerous clades of rhizobia.

Chemotaxis is the systematic movement of an organism through a chemical gradient, and in bacteria operates on the principle of a biased random walk. Bacteria will rotate their flagella to either swim smoothly in a largely straight direction or tumble in place to reorient. The frequency at which cells switch between both modes is dependent on their movement up or down a chemical gradient. If cells are swimming down an attractant gradient (i.e., away from the source of the attractant), they will initiate tumbles more frequently to reorient themselves in the proper direction. If cells are swimming up an attractant gradient (i.e., toward the source of the attractant), the tumbling behavior is suppressed, and smooth swimming is protracted. The net effect of this precept is a gradual translocation to the source of an attractant molecule (Berg, 2003; Parkinson et al., 2015).

The first examination of chemotaxis in *Sinorhizobium* (*Ensifer*) *meliloti* (then called *Rhizobium meliloti*) was in a 1976 study by Currier and Strobel (1976). They tested six different strains of rhizobia for chemotaxis to host legume, non-host legume, and non-legume root exudates. Later, in the 1980s, most publications on the chemotaxis of *S. meliloti* noted attraction to both sugars and amino acids, with the latter appearing to be preferred. However, it is difficult to draw firm conclusions because each of these studies used a different strain, e.g., Ve26, MVII-1, and L5.30 (Burg et al., 1982; Götz et al., 1982; Malek, 1989). In addition, selected studies examined the possibility of chemotaxis to *nod* gene-inducing flavonoids (Caetanoanollés et al., 1988; Dharmatilake and Bauer, 1992). However, these studies lacked context in the form of other classes of test compounds and presented what could be considered comparatively low levels of chemotaxis. Since then, great knowledge was gained about rhizobial chemotaxis, components of the signaling system, and molecular signaling mechanisms (Pleier and Schmitt, 1991; Sourjik and Schmitt, 1996; Sourjik et al., 2000; Rotter et al., 2006). However, follow-up experiments on flavonoid chemotaxis are lacking, and the mechanism of flavonoid sensing in chemotaxis remains undefined.

At outset, the aim of this work was to identify the chemotactic sensor for flavonoids in *S. meliloti*. The most common and best studied mechanism of chemotactic sensing is the direct binding of an attractant molecule to the periplasmic region of a Methyl-accepting Chemotaxis Protein (MCP). As of now, our lab has identified the ligand classes for three of the eight *S. meliloti* MCPs known to be involved in chemotaxis (Meier et al., 2007; Meier and Scharf, 2009; Webb et al., 2014, 2017a,b; Compton et al., 2018). The original goal of this study was particularly salient, especially since, to our knowledge, this would have been the first documented example of a chemotactic sensor for flavonoids in bacteria. We first identified the main flavonoids present in germinating seed exudates of the economically relevant host, alfalfa (*Medicago sativa*). Next, we tested commercially available standards of these compounds in chemotaxis assays. As we tested multiple compounds and experimental conditions, it became apparent that *S. meliloti* was not attracted to any flavonoids identified in alfalfa seed exudates, and therefore, could not confirm previous studies. Based upon these results, we reexamined the relevance of flavonoid chemotaxis in the recruitment of symbiotic rhizobia to the roots of their legume hosts.

## Materials and Methods

### Chemotaxis Assays

Assays for chemotaxis were done in a manner derived from the capillary-based method of Adler (1973). Motile *S. meliloti* RU11/001 and 1,021 cells were grown by inoculating 10 ml of rhizobium basal (RB) medium (RB: 0.1 mM NaCl, 10 μM Na_2_MoO_4_, 6.1 mM K_2_HPO_4_, 3.9 mM KH_2_PO_4_, 1 mM (NH_4_)_2_SO_4_, 1 μM FeSO_4_, 1 mM MgSO_4_, 0.1 mM CaCl_2_, 20 μg/L D-biotin, and 10 μg/L thiamine) overlain on a bromfield medium agar plate (0.4 g/L tryptone, 0.1 g/L yeast extract, 0.45 mM CaCl_2_, and 15 g/L agar; Götz et al., 1982; Sourjik and Schmitt, 1996) overnight at 30°C. Cells were harvested at an OD_600_ of 0.15–0.18, centrifuged at 3,000 *g* for 5 min to remove spent culture, and suspended to an OD_600_ of 0.15. After checking microscopically that greater than 50% of the population were motile, 350 μl of culture was dispensed into a flat glass chemotaxis well. One-microliter microcaps (Drummond Scientific) were sealed at one end and were filled with solution using a vacuum or centrifugation. Chrysoeriol, hyperoside, luteolin-7-O-glucoside, and quercetin-3-O-(6''-acetylglucoside) were acquired from extrasynthese (Genay, France), quercetin, and luteolin from Cayman Chemical (Ann Arbor, MI, United States), and pratensein from Chromadex (Irvine, CA, United States). Flavonoids were dissolved in 100% methanol or dimethyl formamide (DMF) and appropriately diluted in RB. To prepare hyperoside in the absence of a cosolvent, an aliquot was suspended in RB by vortexing and brief incubation at 42°C. After centrifugation, the hyperoside concentration was determined using the molar extinction coefficients ε_259_ = 20,400 M^−1^ cm^−1^ and ε_364_ = 24,500 M^−1^ cm^−1^ (Windholz and Merck and Co., 1983). Capillaries were placed in the chemotaxis wells and left to incubate at room temperature for 2 h. Assays were performed for each concentration in technical triplicate for each of three biological replicates, excepting the methanol dose response experiment, which was done in technical duplicate for each of four biological replicates. The capillaries were broken at the sealed end and their contents were dispensed into RB and appropriately diluted. Dilutions were plated onto tryptone, yeast, calcium chloride plates (TYC: 5 g/L tryptone, 3 g/L yeast extract, 5.9 mM CaCl_2_, and 15 g/L agar) with 0.6 mg/ml streptomycin sulfate. Cells per capillary were calculated by subtracting the number of bacteria that accumulated in a capillary with only RB from each test capillary. Data are also displayed as chemotaxis ratios, which are the quotient of the cells in the test capillary divided by the cells in the reference capillary. This value was included for easier comparison to previous reports that used this method. A capillary containing 10 mM proline or 1 mM sodium acetate was used as a positive control alongside the experiments.

### Flavonoid Quantification From Germinating Seeds

Seed exudates were harvested from Guardsman II variety alfalfa (*M. sativa* L.). For each replicate, 0.1 g seeds were surface sterilized by rinsing four times with sterile water, soaking in 8 ml of 3% H_2_O_2_ for 12 min, and rinsing four times with sterile water. Seeds were left to germinate in 3 ml of sterile water for 24 h at 30°C. At the time of harvesting, an aliquot of seed exudate was examined for contamination microscopically and plated onto TYC. Samples that did not show contamination in the sample or on the plate the next day were flash frozen in liquid nitrogen and stored at −80°C.

For solid phase extraction (SPE), 2.5 ml of 10 separate seed exudate samples were applied to 1 cc Oasis PriME HLB SPE cartridges (Waters, Milford, MA, United States). Each cartridge was washed twice with 1 ml of water and eluted with two 1-ml aliquots of methanol. Flow-through and wash fractions were combined to create the hydrophilic fraction, while the methanol elutions were combined to create the hydrophobic fraction. Both fractions were concentrated to dryness and stored at −20°C. For capillary assay experiments and mass spectrometry analysis, both fractions were suspended in water to five-times their original concentration. When used for capillary assays, fractions and raw exudates were mixed with water and 5-fold concentrated RB to achieve a final experimental concentration of 0.8-fold exudate and 1-fold RB. Since the raw, unfractionated exudates could not be concentrated, 0.8-fold was the highest concentration of raw exudates that could be utilized.

### Mass Spectrometry of Seed Exudate for Flavonoid Profiling

Seed exudates were prepared for analysis by dilution in methanol with 0.1% formic acid (1:1 v/v), sonication in a water bath for 10 min and centrifugation at 13,000 *g* for 10 min. Sample analysis was performed on a Synapt G2-S high resolution Q-TOF mass spectrometer (Waters Corp., Milford, MA, United States) interfaced with an Acquity I-class ultra-performance liquid chromatography (UPLC; Waters Corp., Milford, MA). Mobile phases were 0.1% formic acid (A) and 0.1% formic acid in acetonitrile (B). The flow rate was 0.2 ml/min, and the 20-min elution gradient was: initial 1% B, 0.5 min hold 1% B, gradient to 40% B at 12 min; gradient to 90% B at 17.5 min, 18 min hold at 90% B, and 19 min return to initial conditions. Two microliters of sample was injected onto a Waters BEH C^18^ 1.7 μm, 50 × 2.1 mm column (Waters Corp., Milford, MA) held at 35°C. The mass spectrometer was operated in negative mode under high resolution and MSE settings with a mass scan range of 50–1,800. Instrument parameters were capillary voltage 1.5 kV, source temperature 125°C, sampling cone 30 V, source offset 80, desolvation temperature 350°C, desolvation gas 500 L/h, cone gas 50 L/h, and nebulizer gas 6 bar. The cycle time was 0.2 s, and collision energy was set at 4 eV for low energy scans and ramped from 20 to 40 in the high energy scans. Leucine enkephlan (Waters Corp., Milford, MA) was continuously infused into the source at 5 μl/min and analyzed at 20 s intervals for real-time mass correction.

Data visualization and analysis was performed with MassLynx v 4.2 (Waters Corp., Milford, MA). Peaks corresponding to potential flavonoids were identified in the high energy scan data by searching spectra for aglycone masses related to known flavonoids. Low energy scan data were then used to determine the precursor species and tentative identifications assigned based upon literature and database searches. Authentic standards were purchased and analyzed with the conditions previously described. Assignment of flavonoid identity was based upon standards and seed exudate providing the same mass, retention time, and high energy mass fragments. Analysis of seed exudate fractions following SPE was performed in the same manner as described above for flavonoid profiling.

### Mass Spectrometry of Seed Exudates for Flavonoid Quantification

Quantification of flavonoids was performed on a Shimadzu 8060 triple quadrupole mass spectrometer (Shimadzu Corp., Kyoto, Japan) interfaced with a Shimadzu Nextera UPLC (Shimadzu Corp., Kyoto, Japan). The flow rate was 0.4 ml/min, and the gradient composition was as follows: initial 40% B, gradient from 0.5 to 4 min 90% B, and 5 min return to initial composition. Five microliters were injected onto a Waters BEH C_18_ 1.7 μm, 2.1 × 50 mm (Waters Corp., Milford, MA) held at 40°C. The mass spectrometer was operated in positive ionization, and multiple reaction monitoring (MRM) were developed based upon compound specific transitions (Table 1). Standards were analyzed at concentrations from 1 to 1,000 ng/ml to generate calibration curves. Data were analyzed with Lab Solutions software v 5.93 (Shimadzu Corp., Kyoto, Japan).

**Table 1:** Levels of selected flavonoids from seed exudates.

Flavonoid	MRM transition	ng/seed	pmol/seed
Chrysoeriol^†^	301.05 > 286.00	5 ± 1	16 ± 4
Hyperoside	465.00 > 303.05	2,455 ± 32	4,963 ± 66
Luteolin^†^	287.15 > 153.05	38 ± 9	133 ± 31
Luteolin-7-glucoside	449.10 > 287.00	52 ± 43	117 ± 96
Quercetin^*^	303.15 > 153.15	<LOQ	<LOQ

Five flavonoids were quantified in alfalfa seed exudates using ultra-performance liquid chromatography-mass spectrometry in MRM, positive ion mode. Seed exudates were harvested by incubating surface-sterilized alfalfa seeds in water for 24 h and enriched for flavonoids by solid phase extraction. Values are the means and SDs from six independent replicates.

† Indicates *nod* gene-inducing flavonoid in *S. meliloti*.

* LOQ, limit of quantification (0.5 ng/seed).

## Results

### Identification and Quantification of Flavonoids in Alfalfa Seed Exudates

To identify abundant and symbiotically relevant flavonoid species in germinating alfalfa (*M. sativa*) seed exudates, we utilized a purification scheme (C_18_ solid phase extraction) reported previously for alfalfa seed exudates (Hartwig et al., 1990a). Five flavonoids were identified in this prior work, all related to luteolin, namely, chrysoeriol (3'-methoxyluteolin), luteolin, luteolin-7-O-glucoside (cynaroside, L-7-G), 5-methoxyluteolin, and 3'-5-dimethoxyluteolin (Supplementary Figure S1). Trace amounts of apigenin and 4'-7-dihydroxyflavone were also reported. This purification scheme was coupled to a metabolomics profiling platform (ultra-performance liquid chromatography-quadrupole time of flight mass spectrometry, UPLC-QTOF MS) to assess the flavonoid profile in detail.

Chrysoeriol, luteolin, and L-7-G were confirmed to be present in the seed exudates based upon comparison to authentic standards. The other methoxylated luteolins (3',5-dimethoxyluteolin and 5-methoxyluteolin), were tentatively identified based upon the parent mass and reported fragmentation patterns (7) but were not confirmed with authentic standards. The most abundant peak observed in the exudate LC-MS chromatograms was hyperoside (quercetin 3-O-β-D-galactopyranoside), which exhibited an identical retention time and fragmentation pattern to an authentic standard.

We next focused on several unknowns that contained fragmentation patterns indicative of flavonoids. Quercetin was found to be present in trace amounts, as was an apparent acetylated quercetin glycoside with a (M-H)^−^ mass of 505.0977 m/z. Quercetin-3-O-(6-acetylglucoside) was a possible candidate, but while the fragmentation patterns were similar, the retention times did not match, leaving the compound as a putative acetylated hexosyl flavonoid. While we confirmed the presence of trace levels of apigenin, we did not detect 4'-7-dihydroxyflavone.

The five flavonoid species we have confirmed in seed exudates, namely, chrysoeriol, hyperoside, luteolin, L-7-G, and quercetin do not represent the entire flavonoid profile of alfalfa seed exudates. However, these five compounds are representative of the total flavonoid pool, and can be classified by their functional groups into flavones (luteolin and chrysoeriol) and flavonols (quercetin) and their glycosylated variants (L-7-G and hyperoside; Supplementary Figure S1). Inasmuch, we next quantified the five identified flavonoids in seed exudates on a per seed basis. Hyperoside was the most abundant flavonoid, followed by luteolin, luteolin-7-glucoside, and chrysoeriol. Quercetin was at or below the limit of quantification (LOQ; Table 1). The ratio of hyperoside, luteolin, L-7-G, and chrysoeriol in alfalfa seed exudates on a per seed basis, was determined to be 200:5:5:1.

*Sinorhizobium meliloti* did not exhibit chemotaxis to alfalfa-derived flavonoids and is unaffected by cosolvents. The capillary assay is the gold standard for the quantification of bacterial chemotaxis responses. The five flavonoids detected in seed exudates along with an additional isoflavone (pratensein) were tested in the capillary assay using motile cells of the *S. meliloti* wild-type strain RU11/001. We chose growth conditions that had been determined previously to result in optimal motility and expression of the chemotaxis machinery in *S. meliloti* (Rotter et al., 2006; Meier et al., 2007). Methanol was utilized at a final concentration of 4% (v/v), because the hydrophobic nature of the flavonoids required an organic cosolvent. Therefore, a control with 4% methanol in RB was also tested. Methanol at this concentration did not affect the motility of *S. meliloti* cells for the duration of the experiments, as evaluated by microscopic observation.

**Figure 1:**
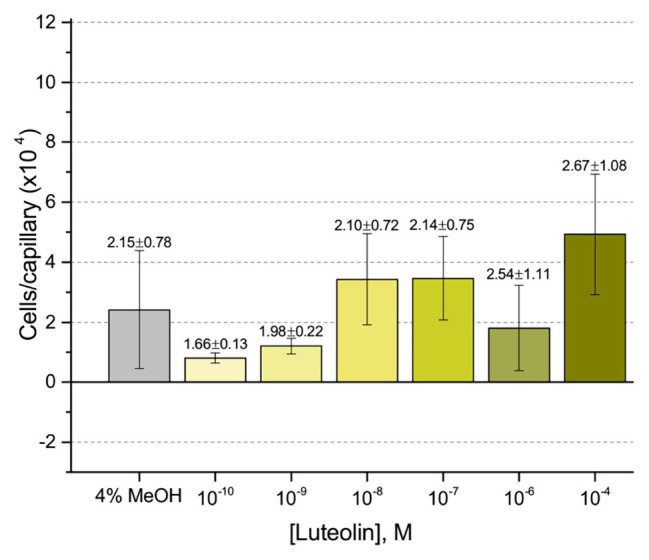
Capillary chemotaxis assay of *Sinorhizobium meliloti* to luteolin and methanol. Each concentration of luteolin tested included 4% methanol in the attractant solution. The bars are the means and SD of three biological replicates in which the number of cells that accumulated in a reference capillary was subtracted from that of the test capillary. The numbers above the bars represent the means and SDs of the chemotaxis ratio, where the number of cells that accumulated in the test capillary were divided by the number of cells that accumulated in a reference capillary.

**Figure 2:**
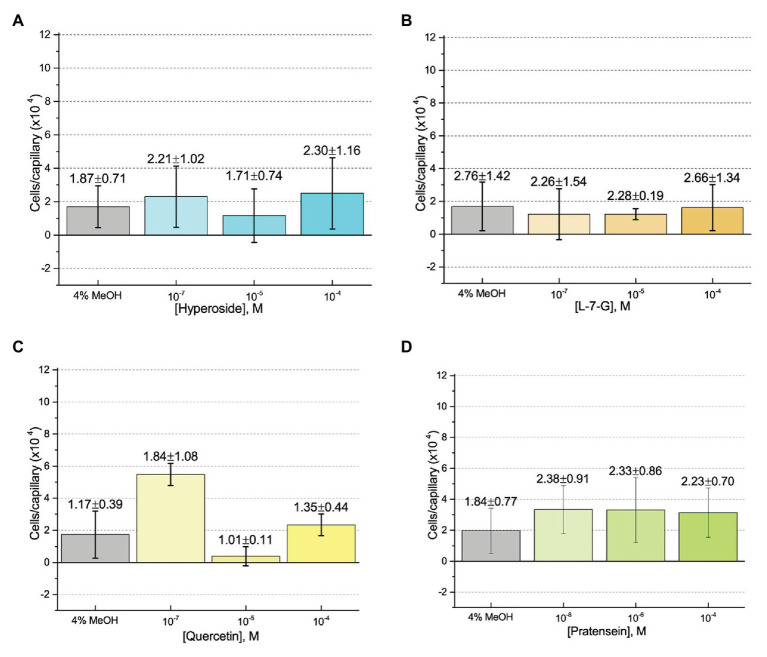
Capillary chemotaxis assays of *S. meliloti* to flavonoids in methanol. Each concentration of flavonoid tested included 4% methanol in the attractant solution. The bars are the means and SD of three biological replicates in which the number of cells that accumulated in a reference capillary was subtracted from that of the test capillary. The numbers above the bars represent the means and SDs of the chemotaxis ratio, where the number of cells that accumulated in the test capillary were divided by the number of cells that accumulated in a reference capillary. **(A)** Hyperoside; **(B)** Luteolin-7-glucoside; **(C)** Quercetin; and **(D)** Pratensein.

**Figure 3:**
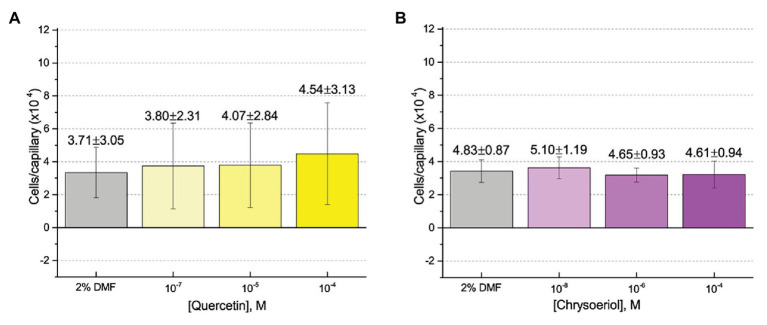
Capillary chemotaxis assays of *S. meliloti* to flavonoids in dimethyl formamide (DMF). Each concentration of flavonoid tested included 2% DMF in the attractant solution. Values are the means and SDs of three biological replicates in which the number of cells that accumulated in a reference capillary was subtracted from that of the test capillary. The numbers above the bars represent the means and SDs of the chemotaxis ratio, where the number of cells that accumulated in the test capillary were divided by the number of cells that accumulated in a reference capillary. **(A)** Quercetin; **(B)** Chrysoeriol.

First, we assessed the major *nod*-gene inducing flavonoid luteolin at six concentrations between 10^−4^ and 10^−10^ M based on concentrations used in previous reports of chemotaxis to this compound (Caetanoanollés et al., 1988; Dharmatilake and Bauer, 1992). The number of cells in capillaries containing luteolin did not differ significantly from the control capillary (Figure 1). The same lack of chemotaxis was observed for four additional flavonoids at three different concentrations between 10^−4^ and 10^−8^ M (Figures 2A–D). Chrysoeriol could not be assayed using the cosolvent methanol because of its poor solubility in this solvent. Therefore, we decided to use DMF at a final concentration of 2% (v/v) as an alternative. In addition, we repeated the capillary assays with quercetin using DMF as cosolvent. Similar to the previous results, a small, but insignificant positive chemotaxis response was observed to the various concentrations of chrysoeriol and quercetin; however, they were indistinguishable from the cosolvent control (Figures 3A,B). In conclusion, we did not observe chemotaxis to any of these flavonoids at any of the tested concentrations.

**Figure 4:**
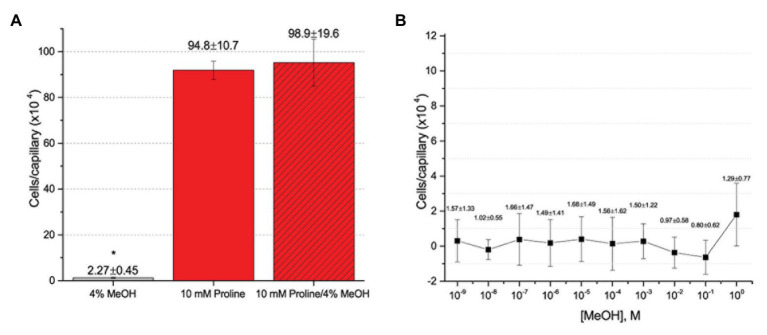
Capillary chemotaxis assays testing the effect of methanol on *S. meliloti* chemotaxis. **(A)** Comparison of chemotaxis to 4% methanol, 10 mM proline, and 10 mM proline + 4% methanol. The bars are the means and SD of three biological replicates in which the number of cells that accumulated in a reference capillary was subtracted from that of the test capillary. The asterisk denotes *p* < 0.0008 using Student’s *t*-test **(B)**. Dose response curve to methanol. The means and SDs were calculated with four biological replicates performed in technical duplicate. The numbers above the bars represent the mean and SD of the chemotaxis ratio, where the number of cells that accumulated in the test capillary were divided by the number of cells that accumulated in a reference capillary. Note the difference in scale between **(A)** and **(B)**.

To further assess the effect of the cosolvent methanol, we examined the chemotaxis of *S. meliloti* to one of its strongest and best characterized attractants, proline, in the absence and presence of 4% methanol (Meier et al., 2007; Webb et al., 2014). The capillary assays clearly demonstrated that addition of methanol at a final concentration of 4% did not significantly change the migration of bacteria into the capillary filled with 10 mM proline (Figure 4A). We next considered the possibility that methanol itself served as a weak attractant and tested this by performing a dose-response curve of chemotaxis to methanol between 1 M (approximately 4% by volume at room temperature) and 10^−9^ M. Methanol did not elicit a chemotaxis response above background at any of the concentrations tested (Figure 4B).

**Figure 5:**
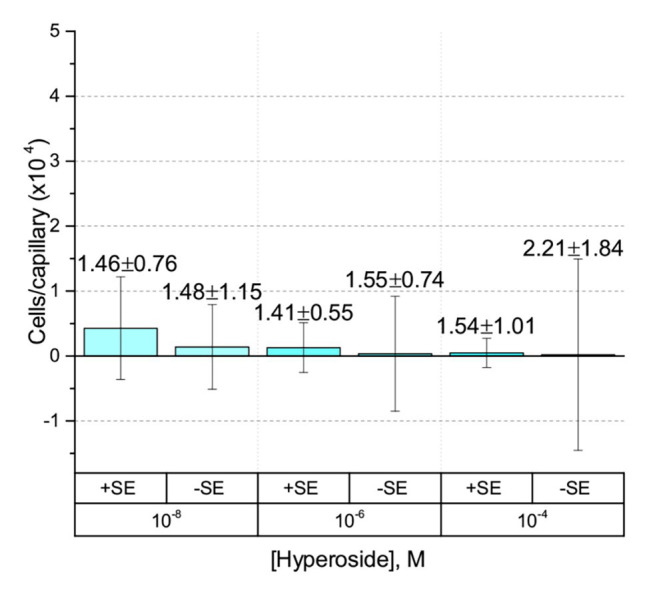
Capillary chemotaxis assays of *S. meliloti* to hyperoside in the absence of cosolvent. Data from cultures grown in the presence of 5% seed exudates (SEs) are indicated with the label + SE on the *x*-axis. Cultures grown without seed exudates are indicated with the label − SE. The bars are the means and SD of three biological replicates in which the number of cells that accumulated in a reference capillary was subtracted from that of the test capillary. The numbers above the bars represent the means and SDs of the chemotaxis ratio, where the number of cells that accumulated in the test capillary were divided by the number of cells that accumulated in a reference capillary. Note that the of the *y*-axis scale is 50% of all preceding experiments.

The only flavonoid detected in alfalfa seed exudates that is soluble in water without the aid of an organic cosolvent is hyperoside, a galactoside of quercetin. We took advantage of this property to obviate the use of a cosolvent in the experiments. No chemotaxis to hyperoside was observed in the absence of a cosolvent (Figure 5). To assess the possibility that the chemotactic response to a flavonoid is inducible, we grew *S. meliloti* cells in the presence of 5% alfalfa seed exudate and examined chemotaxis to hyperoside without methanol. The final concentration of hyperoside, the most abundant flavonoid in alfalfa seed exudates, in the growth medium was approximately 4.7 μM. Despite this amendment, no chemotaxis to hyperoside was detected (Figure 5).

*Sinorhizobium meliloti* is attracted to the seed exudate fractions that are depleted in flavonoids. We wanted to consider taxis to host derived flavonoids in a more direct context pertaining to seed exudates. To achieve this, we harvested seed exudates and fractionated using C_18_ SPE cartridges essentially as described previously (Hartwig et al., 1990a). Flow-through and wash fractions with water were collected and pooled into a hydrophilic, “non-flavonoid” fraction. Adsorbed compounds were eluted with 100% methanol, referred to as the hydrophobic fraction. Confirmation by mass spectrometry analysis showed that flavonoids were enriched in the hydrophobic fraction compared with the hydrophilic fraction, which is likely comprised of amino acids, quaternary ammonium compounds (QACs), and carboxylates (Supplementary Figure S2; Webb et al., 2017a,b; Compton et al., 2018).

**Figure 6:**
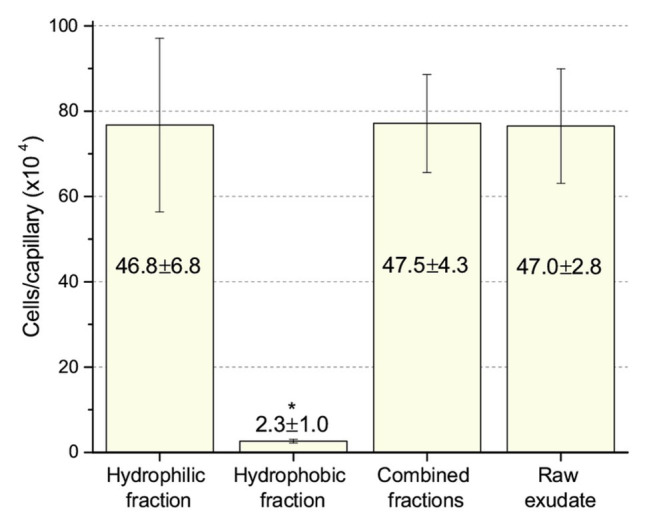
Chemotaxis of *S. meliloti* to fractionated and raw alfalfa seed exudates. Comparison of the chemotactic potential of seed exudate fractions. The hydrophilic fraction is the combined flow-through and water wash. The hydrophobic fraction is the combined methanol washes. Combined fractions are the mixture of hydrophilic and hydrophobic fractions in equal proportion. Raw exudates were not separated by SPE. All fractions were used at 0.8-fold of their original concentration. The asterisk denotes *p* < 0.011 using Student’s *t*-test.

Next, we compared chemotaxis to these two fractions and to the raw, unfractionated exudates. The unfractionated exudates, the hydrophilic fraction, and the combined hydrophilic and hydrophobic fractions all drew approximately 770,000 cells per capillary. The hydrophobic fraction only attracted 26,000 cells per capillary (Figure 6), clearly demonstrating that the dominant chemoattractants are hydrophilic in nature and not flavonoids or other hydrophobic compounds.

### The *S. meliloti* Model Strain, Sm1021, Is Also Unattracted to Flavonoids

**Figure 7:**
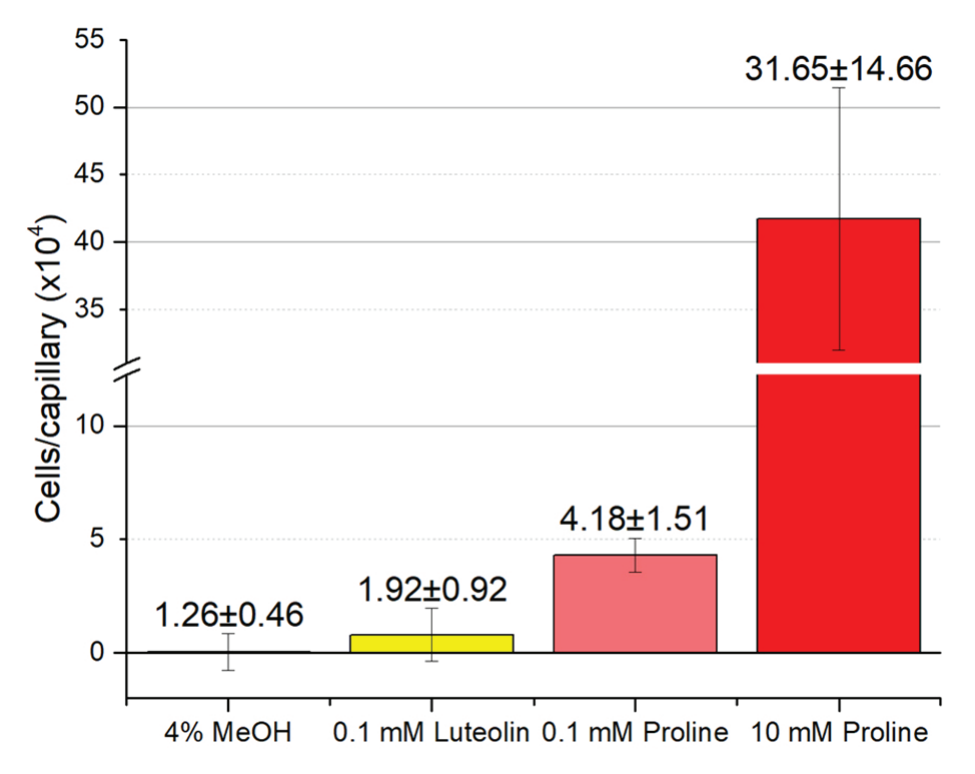
Chemotaxis of *S. meliloti* Sm1021 to luteolin and proline. Comparison of Sm1021 chemoattraction to 4% methanol, luteolin in 4% methanol, 0.1 and 10 mM proline. The bars are the means and SD of three biological replicates in which the number of cells that accumulated in a reference capillary was subtracted from that of the test capillary. The numbers above the bars represent the means and SDs of the chemotaxis ratio, where the number of cells that accumulated in the test capillary were divided by the number of cells that accumulated in a reference capillary.

Although RU11/001 is a model strain for chemotaxis, we decided to also examine the chemotaxis of *S. meliloti* Sm1021 because it is the model strain for studies in the molecular biology of symbiosis. We performed capillary chemotaxis assays with Sm1021 under identical conditions to those used for RU11/001. We tested chemotaxis to luteolin at 10^−4^ M, the highest concentration possible in 4% methanol, alongside 0.1 and 10 mM proline. Similar to the results of RU11/001, proline was a strong attractant at 10, but less so at 0.1 mM, with 417,000 and 43,000 cells per capillary, respectively. These data are lesser than, but congruent with those of the response of RU11/001 to proline (Meier et al., 2007; Webb et al., 2017a). Chemotaxis of Sm1021 to luteolin was again indistinguishable from that of 4% methanol (Figure 7).

## Discussion

### A Retrospective on the History of Rhizobial Chemosensing

Flavonoids are the key chemical signal in the initiation of the symbiosis between legumes and their rhizobial symbionts. This process was conflated with chemotaxis when flavonoids were postulated to also interact with the chemosensory pathways. The first investigation into the roles these compounds play as chemo-attractants in *S. meliloti* was reported in 1988, where it was claimed that *S. meliloti* RCR2011 was attracted to luteolin maximally at a concentration of 10^−8^ M, and that this response disappeared when a large portion of the *nif-nod* region was deleted or individual *nod* genes were interrupted by transposon insertion (Caetanoanollés et al., 1988). Reported values were chemotaxis coefficients of approximately 2; an extremely weak chemotaxis response (Götz et al., 1982). In addition, none of the chemotaxis response curves showed the SE or CIs, making it difficult to compare these responses to the background. Furthermore, the authors did not attempt to recover the wild-type response by complementation (Caetanoanollés et al., 1988). A follow-up study examined the chemotaxis to luteolin, two other host-derived flavonoids, and a chalcone. Unfortunately, and as stated by the authors, the presented data were inconsistent and varied on a day-to-day basis. Nevertheless, *S. meliloti* was concluded to exhibit a chemotaxis to these compounds, though how robust this response was might not have been fully appreciated at the time (Dharmatilake and Bauer, 1992).

### The Chemotaxis Response to Flavonoids Was Also Investigated in Other Rhizobial Species

Aguilar et al. (1988) examined the chemotaxis of *Rhizobium leguminosarum* bv. *phaseoli* RP8002 to a number of sugars and phenolics compounds in addition to the flavonoids luteolin, apigenin, and naringenin. These experiments demonstrated chemotaxis to luteolin and apigenin, and the response to these compounds was comparable in magnitude to the response to xylose and several phenolic compounds. Importantly, it was clear from these experiments that the responses were significantly above background (Aguilar et al., 1988). Using *Bradyrhizobium japonicum* USDA110 as a model organism, Barbour et al. (1991) tested the contribution of numerous compounds identified in soybean exudates, including flavonoids to chemotaxis. This study revealed that *B. japonicum* is most attracted to carbon sources such as succinate, glutamate, and malonate, but not to the flavonoids luteolin, daidzein, daidzin, and genistein. The effect of transposon insertions in several *nod* genes on chemotaxis to genistein and seed exudates was tested, but no change was observed (Barbour et al., 1991). The lack of chemotaxis to flavonoids in *B. japonicum* corroborated the findings of Kape et al. (1991).

While the above studies appear contradictory in nature, only the work of Barbour et al. (1991) incorporated multiple attractants and utilized relatively stringent statistics. Based on experiments using fractionated seed exudates, the authors aptly concluded that “the primary chemotactic components and the primary [*nod* gene] inducing components are chemically separate” (Barbour et al., 1991).

As it stands, the chemotaxis of rhizobia to flavonoids is widely accepted, stated in numerous reviews and textbooks (Subramanian et al., 2007; Maj et al., 2010; Oldroyd et al., 2011; Abdel-Lateif et al., 2012; Hassan and Mathesius, 2012; White et al., 2012; Liu and Murray, 2016). This is an appealing conclusion given that one or a few highly specific molecules could be responsible for both the recruitment of rhizobial symbionts and induction of their symbiotic pathways. In hindsight, if these studies compared the chemotactic potency of flavonoids to other attractants such as amino acids, flavonoids would not be regarded as significant contributors to the recruitment of rhizobia to host plants.

### A Second Look at Chemotaxis to Flavonoids

Our current study provides an in-depth analysis of the importance of host-derived and symbiotically relevant flavonoids in the attraction of *S. meliloti* to its plant host (Table 1). We performed concentration-dependent chemotaxis assays with *S. meliloti* using four flavonoid aglycones (chrysoeriol, luteolin, pratensein, and quercetin) and two single hexosyl glycones (hyperoside and luteolin-7-glucoside) and did not detect chemotaxis to any of these compounds. Of principal importance is the comparison of our results with previous data on luteolin chemotaxis, as it is the only flavonoid tested by all previous reports (Aguilar et al., 1988; Caetanoanollés et al., 1988; Barbour et al., 1991; Dharmatilake and Bauer, 1992). While we observed similar chemotaxis ratios to previous reports (about 2–3), the negative control unequivocally indicates that this is not due to the luteolin (Figure 1; Caetanoanollés et al., 1988; Dharmatilake and Bauer, 1992). The values of chemotaxis we measured to flavonoids were above the background (reference capillaries containing only buffer), but not distinguishable from the methanol or DMF cosolvent control (Figures 1–3). Since chemotaxis to 10 mM proline was not inhibited by the addition of methanol, the cosolvent does not appear to interfere with the bacterium’s ability to sense and swim to attractants (Figure 4). Methanol at a concentration of 4% attracts a certain number of cells but is not an attractant at any concentration below that (Figure 4B). As to the cause of the chemoattraction to methanol and DMF, we propose that at approximate concentrations of 1.2 and 0.27 M, respectively, these solvents perturb the chemotaxis signaling system, potentially *via* membrane disruption (Vaknin and Berg, 2006). We next sought to address the possibility of synergism between flavonoids. To obtain a cocktail that best mirrors what the rhizobia would encounter in the presence of a host, we performed a fractionation of seed exudates, obtaining a hydrophilic fraction, and a hydrophobic, flavonoid-containing fraction. Chemotaxis assays to this flavonoid-enriched sample only showed a modest accumulation of cells, similar to that obtained with individual flavonoids. These data also revealed that the hydrophilic fraction is responsible for 100% of the chemotactic potential of raw seed exudates (Figure 6). This information serves as evidence that the best chemoattractants are water-soluble, or at least poorly retained on a reversed phase SPE unit with water as the eluent. Assays were performed with *S. meliloti* cells grown under optimal motility and chemotaxis conditions and were the same as those used to characterize the mechanisms of chemotaxis to amino acids, QACs, and small monocarboxylates (Webb et al., 2014, 2017a; Compton et al., 2018). Examinations of the regulation of motility and chemotaxis in *S. meliloti* allowed us to identify optimal conditions for this behavior to be examined, such as culturing methods, media, growth phase, and cell density (Sourjik et al., 2000; Rotter et al., 2006; Meier et al., 2007). These conditions were used for all subsequent characterizations of *S. meliloti* chemotaxis, including this study. We chose the strain RU11/001, a highly motile derivative of strain MVII-1, for the present study because it is a model for the chemotaxis systems in rhizobia (Kamberger, 1979; Wibberg et al., 2013). In addition, this strain is capable of forming effective nodules with alfalfa (Meier, 2007). We also included chemotaxis assays of the model symbiont *S. meliloti* strain Sm1021. However, its chemotactic behavior was largely the same as RU11/001 except for a reduced response to 0.1 mM proline.

It should be mentioned here that it is practically impossible to prove a negative. The use of statistics such as *t*-tests only allows us to make the claim that our methods and experimental conditions cannot resolve a difference between treatments, such as a negative control and a concentration of a putative attractant. They do not have the power to claim that the existence of a phenomenon is disproven by the absence of its observation. Directly put, while we do not confirm any evidence of chemoattraction to flavonoids, this does not disprove the existence of chemotaxis to flavonoids overall, in other bacterial species, under other conditions, or using mechanisms other than flagellar motility. That in mind, we feel that the following lines of evidence make this phenomenon less feasible than it initially appears.

### Evidence Against the Possibility of Flavonoid Chemotaxis in *S. meliloti*

The fact that flavonoid aglycones are at best sparingly soluble in aqueous solution is a clue to their function. If released into the spermosphere and rhizosphere without a glycone group, the poor solubility will restrict transport to a small area close to the release site. Using a hydrophobic molecule as an inducer of symbiosis is logical, because a molecule that diffuses too far from the appropriate location on the host would cause spurious and non-productive symbiotic elicitations (Shaw and Hooker, 2008). An effective chemoattractant, however, needs to form a robust, long-distance gradient for a cell to follow (Futrelle and Berg, 1972). This disparity in requirements makes finding a molecule that would effectively fulfill both roles problematic.

The odds of any individual bacterium seeding a nodule are minuscule (Denison and Kiers, 2011). The sheer density of all bacteria (which can approach 10^8^ cells per gram of soil) around a plant’s roots is in great excess to all potential sites of nodule formation (Torsvik et al., 1990; Roesch et al., 2007; Raynaud and Nunan, 2014). Most nodulation-competent rhizobia in the rhizosphere would exist elsewhere than at the tips of root hairs – the location of nodulation. As an alternative, the rhizobia that do not nodulate their host, along with the vast majority of other resident microbiota, can reasonably expect to survive on the exudates from the plant roots. It has been well-documented that the rhizosphere is far richer in carbon than the surrounding bulk soil (Chiu et al., 2002; Jia et al., 2015). Essentially, all plants release root exudates, and the propensity to seek areas rich in carbon and nitrogen sources would allow rhizobia to acquire nutrients regardless of the source plant. This may be a superior survival strategy compared to seeking out a specific host plant for the express purpose of a low percentage chance of nodulation. Therefore, a flavonoid chemotaxis system would not be practical for the majority of circumstances rhizobia encounter, and since numerous other attractants are released from root hair tips, the bacterium would inherently swim to that location anyway, making a flavonoid sensing system redundant.

Our lab has 2 decades of experience in the study of multiple facets of rhizobial chemotaxis (Sourjik et al., 2000; Rotter et al., 2006; Meier et al., 2007; Meier and Scharf, 2009; Dogra et al., 2012; Shrestha et al., 2018). We have so far characterized the molecular sensing mechanisms of three different classes of chemoattractants. In particular, L-proline, sensed by McpU, has a chemotaxis coefficient of 100 (Webb et al., 2014). Stachydrine and other QACs sensed by McpX elicit chemotaxis coefficients around 80 (Webb et al., 2017a). McpV directly senses small monocarboxylates, but its ligands have chemotaxis coefficients of only 4 (Compton et al., 2018). While it is unwise to make direct comparisons between different strains, we note that Caetanoanollés et al. (1988) reported a chemotaxis ratio of 2 to luteolin using their techniques and conditions. Although this result agrees with our data on chemotaxis to flavonoids, we do not and cannot claim that taxis to flavonoids is distinguished from taxis to the methanol cosolvent (Figures 1, 2). Given the magnitudinous differences in chemotaxis coefficients, we conclude that amino acids and QACs are the primary metabolites plant hosts can use to recruit motile *S. meliloti*. Amino acids are about 10-fold more abundant by mass than the QACs in alfalfa seed exudates, indicating that the amino acids are the most important recruitment signals. This conclusion is supported by chemotaxis experiments with synthetic mixtures of the amino acid spectrum in seed exudates, which showed that the amino acid fraction alone can elicit 23% of the response to whole seed exudates (Webb et al., 2017b). In our hands, the signal to noise ratio of the capillary assay makes attractants with chemotaxis ratios below 3 difficult to identify with statistical significance. However, even if an attractant with such a low level of attraction could be accurately identified, its significance would still be dwarfed by far more potent attractants, such as QACs and amino acids. Our current understanding of chemotaxis in *S. meliloti* indicates that the amino acids are the most important known attractants because of the strong chemotaxis response and their high abundance in seed exudates (Webb et al., 2014, 2016, 2017b). The QACs are almost as relevant to host seed sensing; while they elicit a similar chemotaxis response, QACs are less abundant than the amino acids (Webb et al., 2017a). Carboxylates are chemically highly diverse, and their presence has been noted in certain plant exudates, but they do not appear to be priority attractants for *S. meliloti* (Garcia et al., 2001; Compton et al., 2018).

The paradigm of flavonoids acting as attractants for rhizobia was established several decades ago. This information became central to the thinking and models in the field of how the rhizobium-legume mutualism is initiated and established. A reexamination of past data in light of current knowledge on chemotaxis signaling in combination with the information we presented here suggests that this paradigm is more of a plant-centric fallacy than a significant ecological phenomenon. Going forward, we hope the field acknowledges that the recruitment, culturing, and communication involved between bacteria and plant hosts are dependent on numerous chemical cues and diverse molecular mechanisms.

## Data Availability Statement

All datasets presented in this study are included in the article/Supplementary Material.

## Author Contributions

KC designed experiments, conducted experiments, analyzed data, and wrote the manuscript. SH and RH designed experiments, conducted experiments, and analyzed data. BS designed experiments, analyzed data, and wrote the manuscript. All authors contributed to the article and approved the submitted version.

### Conflict of Interest

The authors declare that the research was conducted in the absence of any commercial or financial relationships that could be construed as a potential conflict of interest.
