# Supplementary material for: An Updated Perspective on Sinorhizobium meliloti Chemotaxis to Alfalfa Flavonoids
Source: Front Microbiol. 2020 Oct 23;11:581482. doi: 10.3389/fmicb.2020.581482 (PMC7644916; doi:10.3389/fmicb.2020.581482)
Supplement: Supplementary file 1 [file Data_Sheet_1.pdf]

SUPPLEMENTAL MATERIALS

An updated perspective on *Sinorhizobium meliloti* chemotaxis to alfalfa flavonoids

K. KARL COMPTON[1], SHERRY B. HILDRETH[1], RICHARD F. HELM[2], BIRGIT E. SCHARF[1]*

[1] Virginia Tech, Department of Biological Sciences, Life Sciences I, Blacksburg, VA 24061
[2] Virginia Tech, Department of Biochemistry, Fralin Life Sciences Institute, VA 24061

List of Figures

Figure S1. Structures of Flavonoids.

Fig. S2. LC-MS Chromatograms in Base Peak Mode. Traces are normalized to relative ion abundance of hyperoside in the original seed exudate. “Fast gradient” LC conditions.

Fig. S3. Selected Ion Monitoring for Luteolin-, Quercetin- and Apigenin-based Compounds.

Fig. S4. Selected Ion Monitoring for Luteolin- and Quercetin-based Compounds.

Apigenin

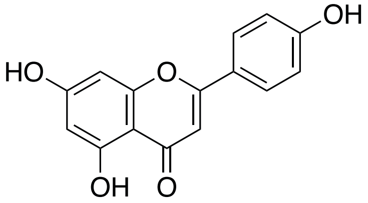

Chemical Formula: $C_{15}H_{10}O_5$
Exact Mass: 270.05282
$[M\text{-}H]^- = 269.0456$

Quercetin

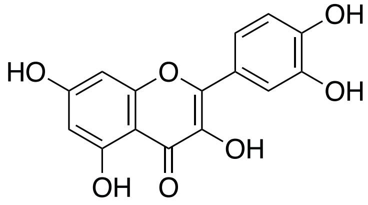

Chemical Formula: $C_{15}H_{10}O_7$
Exact Mass: 302.04265
$[M\text{-}H]^- = 301.0354$

Luteolin

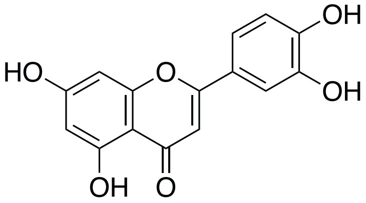

Chemical Formula: $C_{15}H_{10}O_6$
Exact Mass: 286.04774
$[M\text{-}H]^- = 285.0405$

Chrysoeriol
3'-methoxy-luteolin

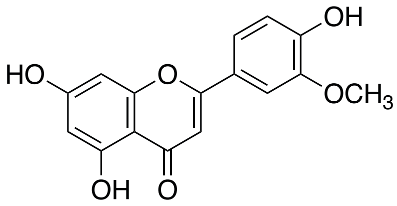

Chemical Formula: $C_{16}H_{12}O_6$
Exact Mass: 300.0634
$[M\text{-}H]^- = 299.0561$

5-methoxyluteolin

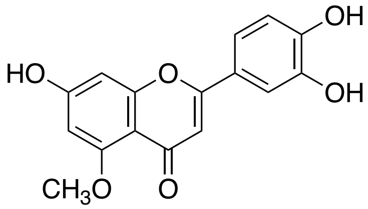

Chemical Formula: $C_{16}H_{11}O_6^-$
Exact Mass: 300.0634
$[M\text{-}H]^- = 299.0561$

3',5-dimethoxyluteolin

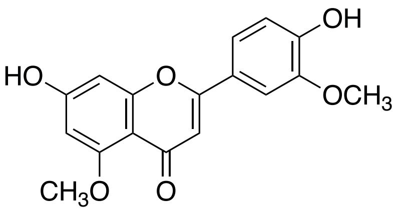

Chemical Formula: $C_{17}H_{14}O_6$
Exact Mass: 314.07904
$[M\text{-}H]^- = 313.0718$

Hyperoside (quercetin 3-galactoside)

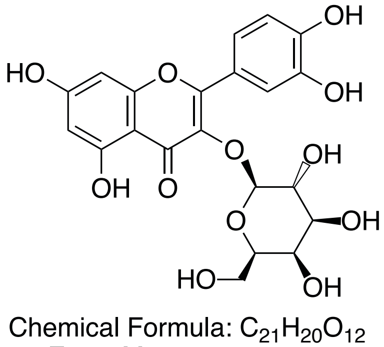

Chemical Formula: $C_{21}H_{20}O_{12}$
Exact Mass: 464.09548
$[M\text{-}H]^- = 463.0882$

Cynaroside (luteolin 7-glucoside)

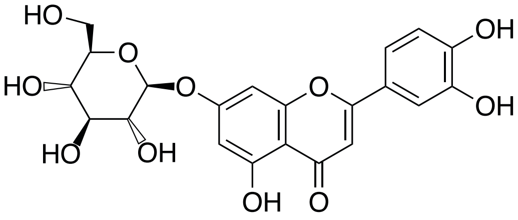

Chemical Formula: $C_{21}H_{20}O_{11}$
Exact Mass: 448.10056
$[M\text{-}H]^- = 447.0933$

Pratensein

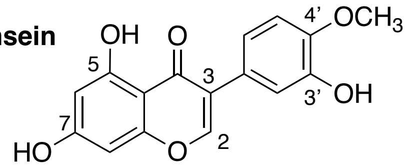

Chemical Formula: $C_{16}H_{12}O_6$
Exact Mass: 300.0634
$[M\text{-}H]^- = 299.0561$

Fig. S1. Structures of Flavonoids.

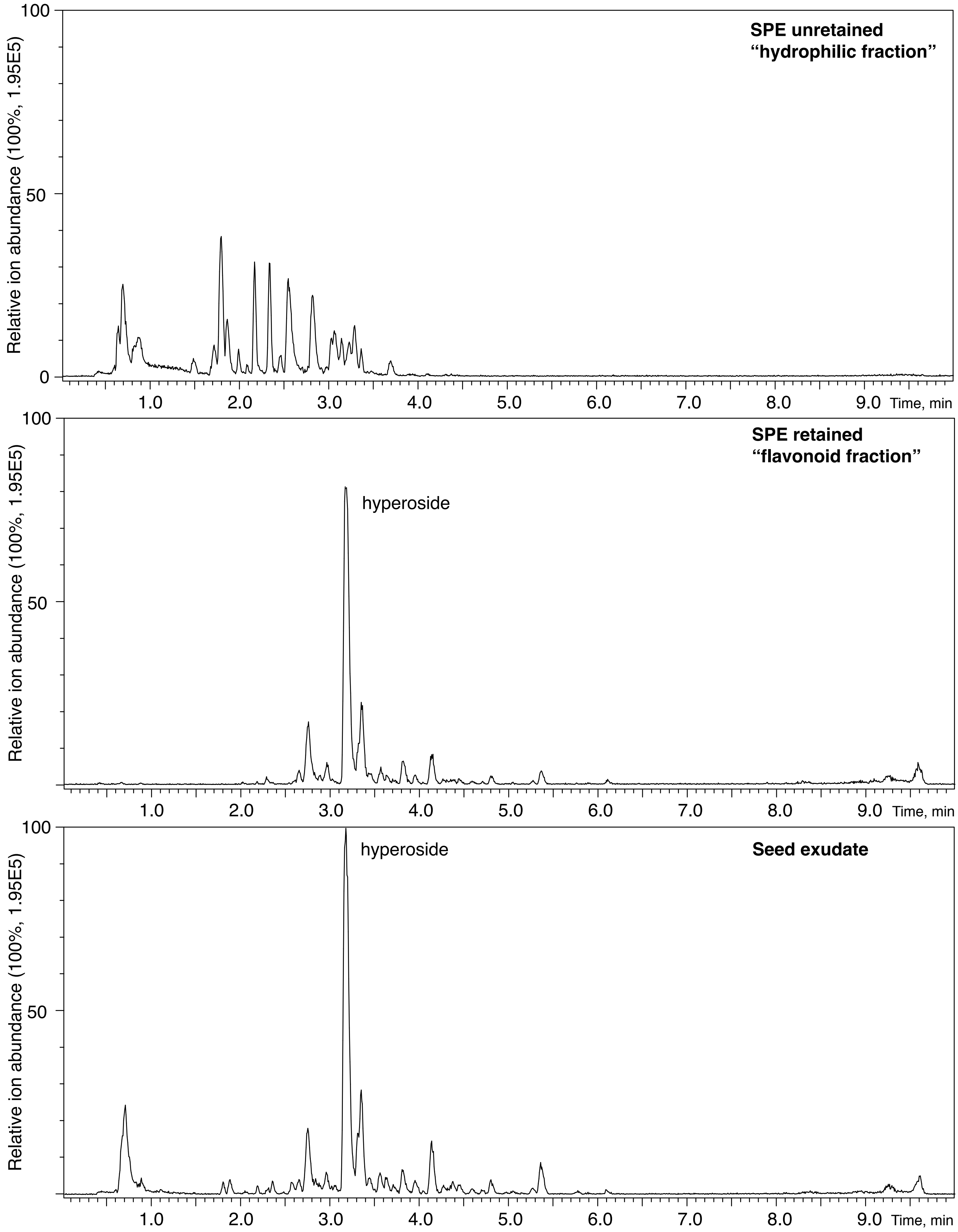

Fig. S2. LC-MS Chromatogram in Base Peak Mode. Traces are normalized to relative ion abundance of hyperoside in the original seed exudate. “Fast gradient” LC conditions. Hyperoside was confirmed with an authentic standard (retention time, parent mass, and fragmentation pattern).

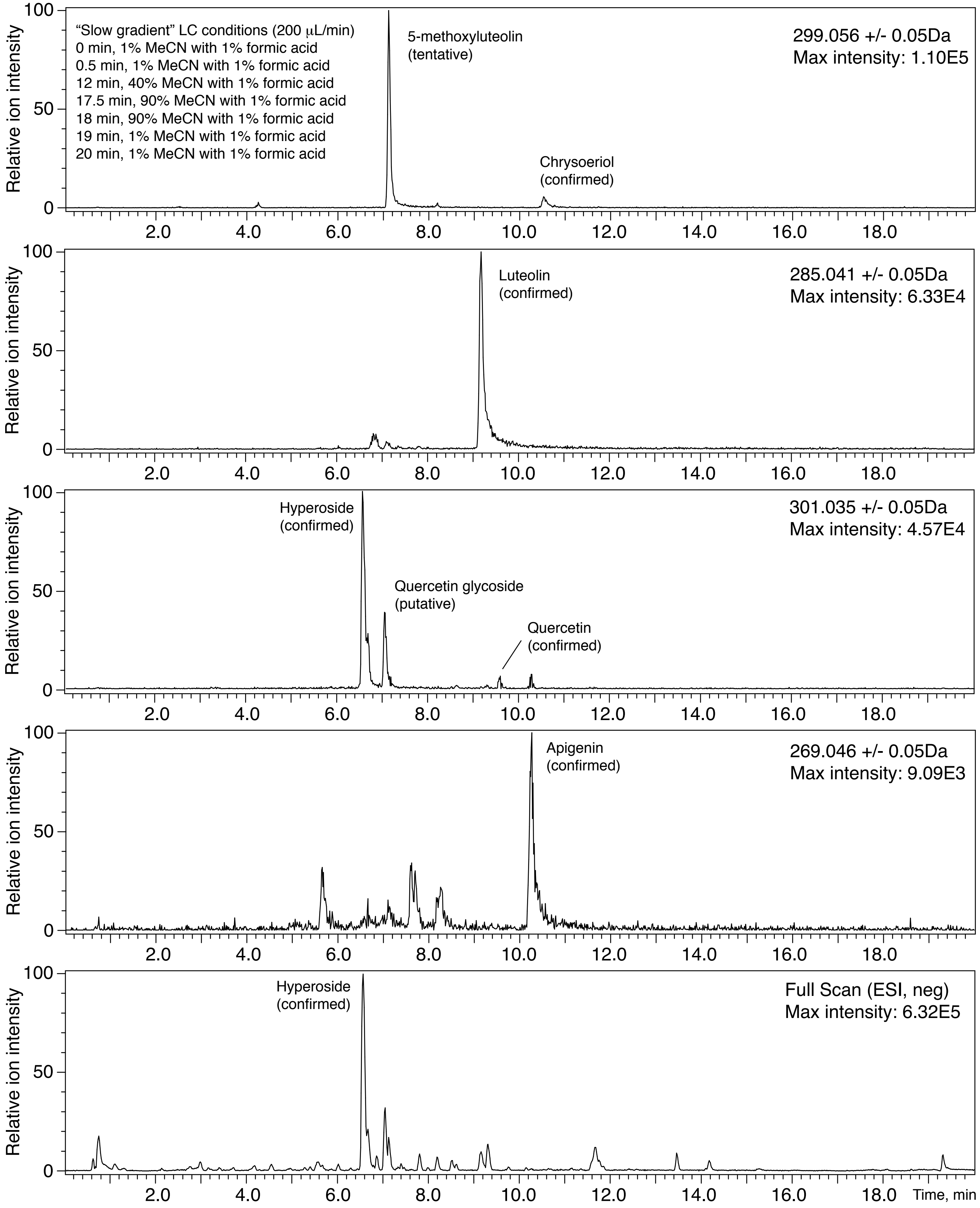

Fig. S3. Selected Ion Monitoring for Luteolin-, Quercetin- and Apigenin-based Compounds. Confirmed: authentic standard, retention time, parent mass, and fragmentation pattern matches. Tentative: previous literature, no authentic standard. Putative: parent mass and fragmentation pattern only.

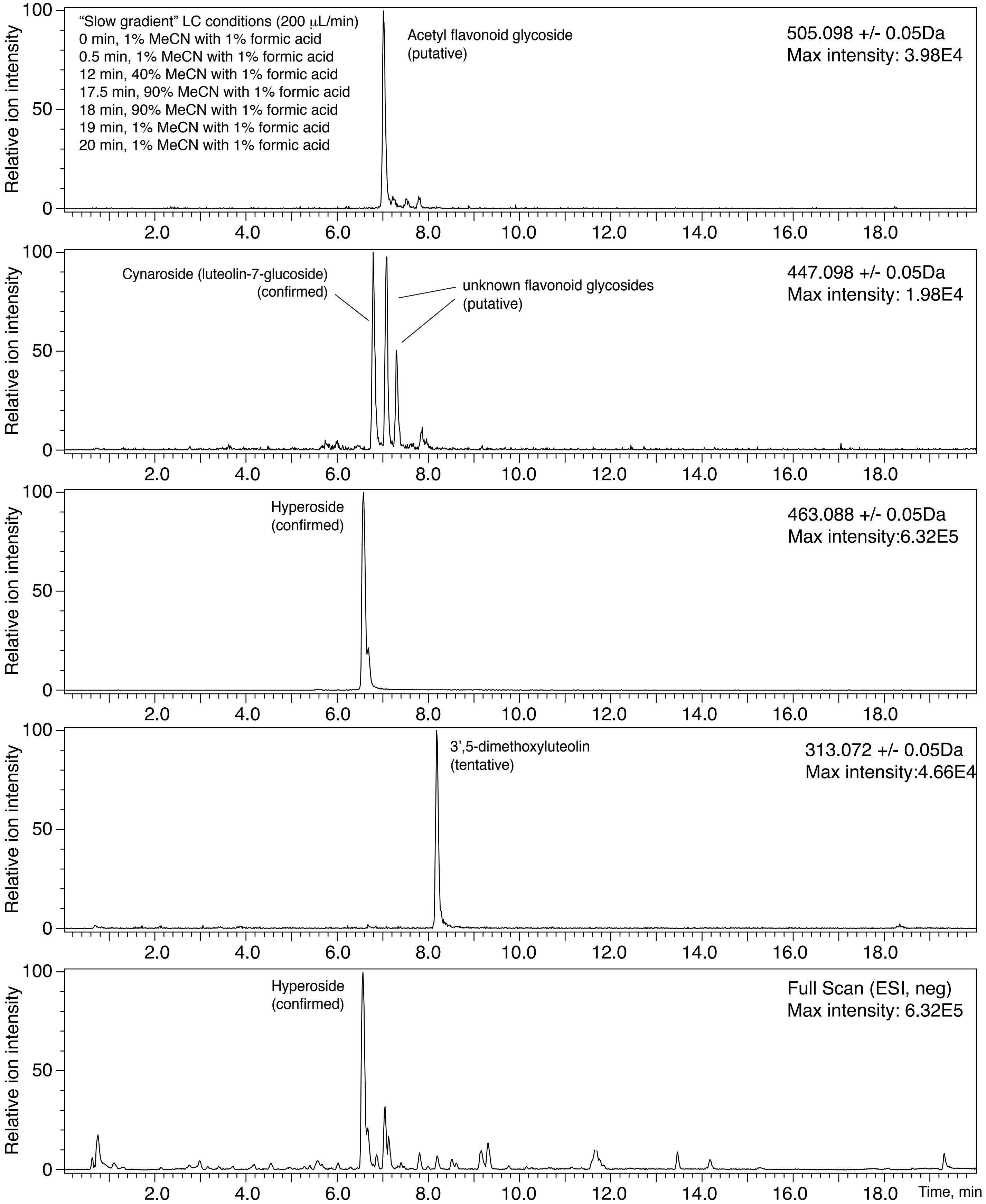

Fig. S4. Selected Ion Monitoring for Luteolin- and Quercetin-based Compounds. Confirmed: authentic standard, retention time, parent mass, and fragmentation pattern matches.Tentative: previous literature, no authentic standard. Putative: parent mass and fragmentation pattern only.
